# Effect of a selective neutrophil elastase inhibitor on mortality and ventilator-free days in patients with increased extravascular lung water: a post hoc analysis of the PiCCO Pulmonary Edema Study

**DOI:** 10.1186/s40560-014-0067-y

**Published:** 2014-12-31

**Authors:** Takashi Tagami, Ryoichi Tosa, Mariko Omura, Hidetada Fukushima, Tadashi Kaneko, Tomoyuki Endo, Hiroshi Rinka, Akira Murai, Junko Yamaguchi, Kazuhide Yoshikawa, Nobuyuki Saito, Hideaki Uzu, Yoichi Kase, Makoto Takatori, Hiroo Izumino, Toshiaki Nakamura, Ryutarou Seo, Yasuhide Kitazawa, Manabu Sugita, Hiroyuki Takahashi, Yuichi Kuroki, Takayuki Irahara, Takashi Kanemura, Hiroyuki Yokota, Shigeki Kushimoto

**Affiliations:** Department of Emergency and Critical Care Medicine, Nippon Medical School, 1-1-5 Sendagi, Bunkyo-ku, Tokyo 113-8603 Japan; Department of Clinical Epidemiology and Health Economics, School of Public Health, Graduate School of Medicine, The University of Tokyo, Tokyo, Japan; Department of Emergency and Critical Care Medicine, Aizu Chuo Hospital, Fukushima, Japan; Department of Emergency and Critical Care Medicine, Nara Medical University, Nara, Japan; Advanced Medical Emergency and Critical Care Center, Yamaguchi University Hospital, Yamaguchi, Japan; Department of Emergency and Critical Care Medicine, Tohoku University Hospital, Miyagi, Japan; Emergency and Critical Care Medical Center, Osaka City General Hospital, Osaka, Japan; Department of Emergency and Critical Care Medicine, Faculty of Medicine, Fukuoka University, Fukuoka, Japan; Division of Emergency and Critical Care Medicine, Department of Acute Medicine, Nihon University School of Medicine, Tokyo, Japan; Shock Trauma and Emergency Medical Center, Tokyo Medical and Dental University Hospital of Medicine, Tokyo, Japan; Department of Emergency and Critical Care Medicine, Nippon Medical School Chiba Hokusou Hospital, Chiba, Japan; Department of Emergency and Critical Care Medicine, Kurume University School of Medicine, Fukuoka, Japan; Critical Care Medicine, Jikei University School of Medicine, Tokyo, Japan; Department of Anesthesia and Intensive Care, Hiroshima City Hospital, Hiroshima, Japan; Advanced Emergency and Critical Care Center, Kansai Medical University Takii Hospital, Osaka, Japan; Intensive Care Unit, Nagasaki University Hospital, Nagasaki, Japan; Intensive Care Unit, Kobe City Medical Center General Hospital, Hyogo, Japan; Department of Emergency and Critical Care Medicine, Kansai Medical University, Osaka, Japan; Department of Emergency and Critical Care Medicine, Juntendo University Nerima Hospital, Tokyo, Japan; Department of Intensive Care Medicine, Saiseikai Yokohamashi Tobu Hospital, Kanagawa, Japan; Department of Emergency and Critical Care Medicine, Social Insurance Chukyo Hospital, Aichi, Japan; Department of Emergency and Critical Care Medicine, Nippon Medical School Tama Nagayama Hospital, Tokyo, Japan; Emergency and Critical Care Medicine, National Hospital Organization Disaster Medical Center, Tokyo, Japan; Division of Emergency Medicine, Tohoku University Graduate School of Medicine, Miyagi, Japan

**Keywords:** Acute lung injury, Extravascular lung water, Pulmonary edema, Pulmonary vascular permeability index, Transpulmonary thermodilution technique

## Abstract

**Background:**

Neutrophil elastase plays an important role in the development and progression of acute respiratory distress syndrome (ARDS). Although the selective elastase inhibitor, sivelestat, is widely used in Japan for treating ARDS patients, its effectiveness remains controversial. The aim of the current study was to investigate the effects of sivelestat in ARDS patients with evidence of increased extravascular lung water by re-analyzing a large multicenter study database.

**Methods:**

A post hoc analysis of the PiCCO Pulmonary Edema Study was conducted. This multicenter prospective cohort study included 23 institutions in Japan. Adult mechanically ventilated ARDS patients with an extravascular lung water index of >10 mL/kg were included and propensity score analyses were performed. The endpoints were 28-day mortality and ventilator-free days (VFDs).

**Results:**

Patients were categorized into sivelestat (*n* = 87) and control (*n* = 77) groups, from which 329 inverse probability-weighted group patients (162 vs. 167) were generated. The overall 28-day mortality was 31.1% (51/164). There was no significant difference in 28-day mortality between the study groups (sivelestat vs. control; unmatched: 29.9% vs. 32.5%; difference, −2.6%, 95% confidence interval (CI), −16.8 to 14.2; inverse probability-weighted: 24.7% vs. 29.5%, difference, −4.8%, 95% CI, −14.4 to 9.6). Although administration of sivelestat did not alter the number of ventilator-free days (VFDs) in the unmatched (9.6 vs. 9.7 days; difference, 0.1, 95% CI, −3.0 to 3.1), the inverse probability-weighted analysis identified significantly more VFDs in the sivelestat group than in the control group (10.7 vs. 8.4 days, difference, −2.3, 95% CI, −4.4 to −0.2).

**Conclusions:**

Although sivelestat did not significantly affect 28-day mortality, this treatment may have the potential to increase VFDs in ARDS patients with increased extravascular lung water. Prospective randomized controlled studies are required to confirm the results of the current study.

**Electronic supplementary material:**

The online version of this article (doi:10.1186/s40560-014-0067-y) contains supplementary material, which is available to authorized users.

## Background

Acute respiratory distress syndrome (ARDS) is a rapid, progressive form of respiratory failure characterized by life-threatening hypoxemia and permeability pulmonary edema [1-3]. Several previous studies have suggested that neutrophil elastase (NE) increases pulmonary vascular permeability, causes proteolysis of pulmonary tissue, and increases production of leukocyte chemotactic factors, which synergistically cause lung injury [1,4]. Previous studies have shown that plasma NE levels correlate with the severity of lung injury in both animal models and human ARDS patients [4,5]. Thus, antagonists or inhibitors of NE synthesis could be effective for treating ARDS. Sivelestat sodium hydrate (Elaspol, Ono Pharmaceutical Co., Ltd., Osaka, Japan) is a specific small-molecule NE inhibitor drug. The effects of this NE inhibitor were demonstrated in various experimental animal models of ARDS [4,6] and also supported by clinical trials [7]. Thus, sivelestat has been approved in Japan and Korea.

However, the effectiveness of sivelestat remains controversial, despite being widely used in Japan for the treatment of ARDS. Two major phase 3 sivelestat trials have been reported to date [8,9]. Tamakuma et al. [9] reported that in a multicenter clinical study, sivelestat contributed to early ventilator weaning in ARDS patients, resulting in earlier transfer to a general ward. However, in the Sivelestat Trial in ALI Patients Requiring Mechanical Ventilation (STRIVE) study [8], sivelestat was not efficacious in patients with ARDS, even in the ventilator-assisted period, and it had no effect on mortality. More recently, positive results were reported following a phase 4 study (postmarket clinical study) in Japan, where both ventilator-free days (VFDs) and 180-day survival rates improved in ARDS patients receiving sivelestat [10]. In contrast, a systematic review and meta-analysis by Iwata et.al [11] concluded that sivelestat was not associated with decreased mortality in ARDS patients. Therefore, although the latest Japanese guidelines for the management of sepsis suggested that sivelestat administration “may be considered” in patients with ARDS [12], its effectiveness remains controversial.

The most reliable pathophysiological feature of ARDS is the development of diffuse alveolar damage (DAD) with increased permeability [13], which results in the accumulation of water in the lungs; this is designated extravascular lung water (EVLW). Although it was difficult to evaluate pulmonary edema caused by acute lung injury (i.e., DAD) quantitatively, recent studies have suggested that transpulmonary thermodilution-derived variables, namely the EVLW index (EVLWi) and the pulmonary vascular permeability index (PVPI), may be informative [14]. Several clinical studies conducted with ARDS patients suggested that both EVLWi and PVPI correlated with disease severity and were independent risk factors of 28-day mortality [15-17]. Thus, these variables have significant clinical implications and may be the key “bridge” for a pathologic-clinical correlation [14,18]. However, no previous sivelestat study has measured these indices.

The aim of the current study was to investigate the effect of sivelestat on mortality and VFDs in ARDS patients with evidence of increased EVLW, by re-analyzing a large multicenter cohort study database.

## Methods

The current study was a post hoc analysis of the PiCCO Pulmonary Edema Study, a prospective cohort study that examined patients with respiratory-distress who were admitted to 23 participating institutions in Japan [16,17,19-22]. This study was approved by the ethics committee of the Nippon Medical School Hospital, and all the other 22 participating institutional ethics committees, and written informed consent was provided by all of the patients’ next of kin.

### Data source and patient selection

The detailed PiCCO Pulmonary Edema study protocols have been described previously [16,17,19-22]. In short, between March 2009 and August 2011, 301 patients with respiratory insufficiency were enrolled in the PiCCO Pulmonary Edema Study. The primary inclusion criteria were as follows: age of >15 years, mechanical ventilation expected to be required for >48 h for acute respiratory failure with a PaO_2_/FiO_2_ ratio of ≤300 mmHg, and bilateral infiltration, as determined by chest radiography. An increase in the EVLWi of >10 mL/kg was used to define pulmonary edema, in accordance with published definitions [19]. The exclusion criteria were as follows: a lapse of over 5 days from the onset of acute respiratory failure; chronic respiratory insufficiency; a history of pulmonary resection, pulmonary thromboembolism, or severe peripheral arterial disease; a cardiac index of <1.5 L/min/m^2^; lung contusion; burns; and other causes rendering the patient unsuitable for evaluation with the transpulmonary thermodilution technique [19].

Assessment of the pathophysiological diagnostic differential for respiratory insufficiency was performed by at least three experts (specializing in intensive care, respiratory disease, and cardiology) who retrospectively determined the pathophysiological mechanism of respiratory insufficiency as being (a) cardiogenic (hydrostatic) pulmonary edema, (b) permeability pulmonary edema (i.e., ARDS), or (c) pleural effusion with atelectasis but no evidence of lung edema secondary to increased hydrostatic pressure or vascular permeability as previously described [19]. The experts carefully scrutinized each patient’s medical history, clinical presentation, and course and the results of their chest computed tomography, radiography, and echocardiography examinations. They also considered the time course of all of the preceding findings, including daily fluid intake, output, and the balance thereof, and the requirement for systemic management and respiratory therapy.

We considered the increased permeability pulmonary edema group (i.e., (b) above) as ARDS [16,19] and included these patients in the current study. Patients who were not administered sivelestat on Day 0 were excluded from the current analysis. The decision to use sivelestat or not was left to the physician in charge of each participating institution. The standard dosage of sivelestat approved in Japan (0.2 mg/kg/h) was administered intravenously after intensive care unit (ICU) admission until it was discontinued based on a clinical decision.

### Variables and endpoint

At the time of enrollment (Day 0), the patient was evaluated with regard to his/her clinical condition, cause of respiratory insufficiency, acute physiology and chronic health evaluation (APACHE) II score, and sequential organ failure assessment (SOFA) score [23,24]. Echocardiography and chest-computed tomography were conducted on the day of enrollment. The patient’s clinical course, including respirator setting, SOFA score, daily fluid intake/output and balance, single-indicator transpulmonary thermodilution-derived variables, and therapeutic interventions were recorded daily. The hospital type was categorized as academic or non-academic. Hospital volume was defined as the number of patients that participated in the current analysis and was categorized into tertiles (i.e., low, medium, and high). The principles and validation of the single-indicator transpulmonary thermodilution-derived variables used in the current study have been discussed in detail elsewhere [14]. The EVLW estimates the amount of water present in the lungs and, thus, the extent of pulmonary edema. The PVPI allows for quantitative differentiation of hydrostatic pulmonary edema from ARDS [19,25] and is strongly correlated with the plasma NE level [26]. The PVPI was calculated as the ratio between the EVLW and the pulmonary blood volume. The EVLW was indexed using predicted body weight (EVLWi; normal range 7.4 ± 3.3 mL/kg) [27-29].

The endpoints used in the current study were 28-day mortality and VFDs [30]. VFDs were defined as the number of days alive and breathing without mechanical assistance during the first 28 days after admission, and patients who died before day 28 were assigned zero VFDs [30].

### Statistical analysis

Data were expressed as mean (standard deviation [SD]) or median (quartile) as appropriate. Continuous variables were compared between the groups using the *t*-test or Mann–Whitney *U*-test as appropriate. Categorical variables were analyzed using the chi-square test or Fisher’s exact test. The patients were divided into two groups: those who received sivelestat and those who did not (control group). Descriptive statistics are presented for all patients, propensity score-weighted (inverse probability of treatment weighting, IPTW) groups, and propensity score-matched groups. The usefulness and details of the propensity score approach in clinical studies have been discussed elsewhere [10,31,32]. To estimate the propensity score, we fitted a regression model for receipt of sivelestat as a function of patient demographics and hospital factors including age, sex, hospital type (academic or non-academic), hospital volume, cause of ARDS (direct or indirect lung injury), APACHE II score, SOFA score, systemic inflammatory response syndrome (SIRS) score, mean arterial pressure, central venous pressure, fluid balance on Day 0, PaO_2_/FiO_2_ ratio, positive end-expiratory pressure (PEEP) level, global end-diastolic volume index (GEDI) on Day 0, cardiac index on Day 0, EVLWi on Day 0, PVPI on Day 0, corticosteroid administration, catecholamine administration, continuous hemodiafiltration therapy, diuretic administration, and polymyxin B hemoperfusion therapy [3,7-12,18,23,24,26,33-52]. One-to-one matched analysis using nearest-neighbor matching and IPTW estimators were performed based on the estimated propensity scores of the patients. A match occurred when a patient in the sivelestat group had an estimated score within 0.25 standard deviations of a patient in the control group. IPTW used weights based on the propensity score to create a synthetic sample in which the distribution of measured baseline covariates was independent of treatment assignment. Weights were defined as W*i* = Z*i*/e*i* + (1 − Z*i*)/1 − e*i*, where Z*i* was an indicator variable denoting whether or not the *i*-th subject was treated, and e*i* denoted the propensity score for the *i-*th subject. An essential component to any propensity score analysis is an assessment of the similarity of baseline covariates (i.e., balancing) between treated and untreated subjects in the matched sample or in the sample weighted by IPTW. We examined balance in baseline variables using standardized differences, where >0.10 was regarded as imbalanced [31,32]. All data were analyzed using SPSS 22.0 for Windows (IBM, Armonk, NY, USA).

## Results

### Patients

**Figure 1:**
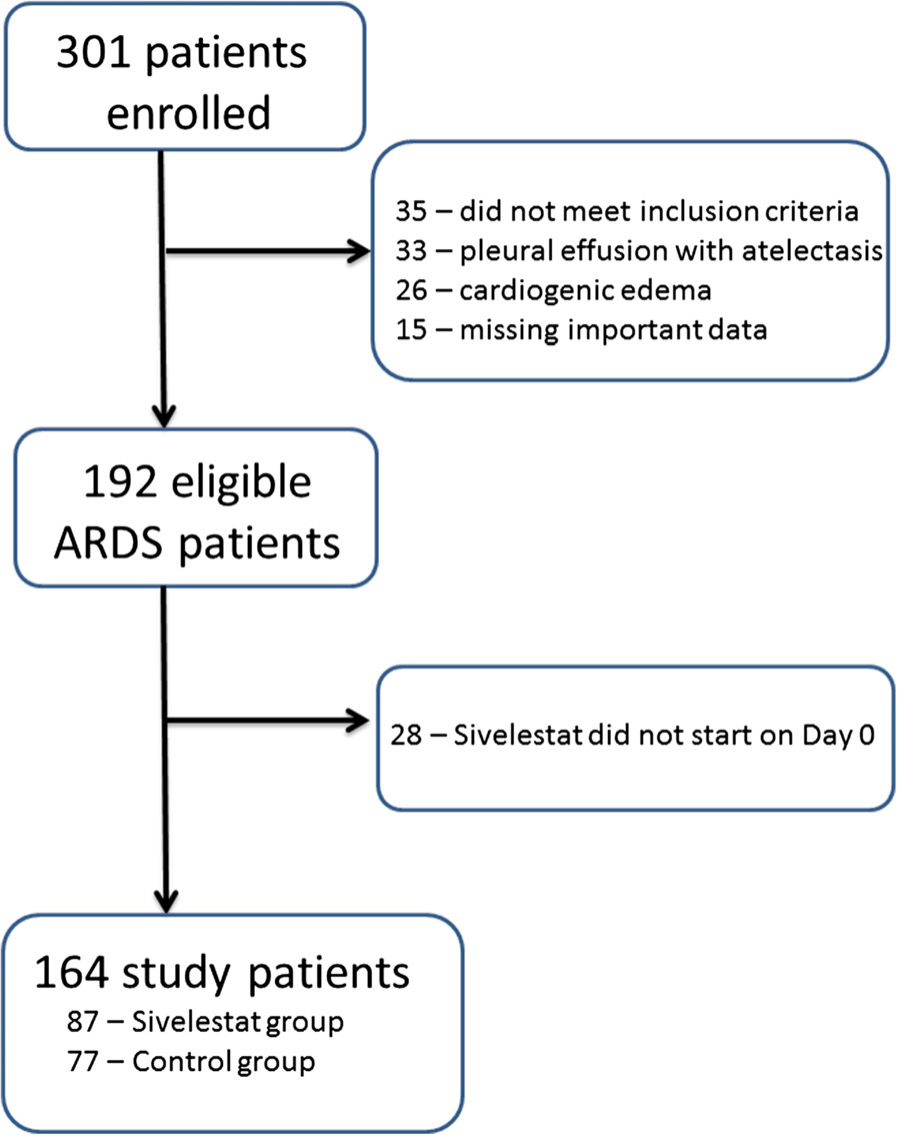
**Flow diagram of patient enrollment.** *ARDS* acute respiratory distress syndrome.

Of the 301 patients initially diagnosed with acute respiratory failure, 192 met the primary inclusion criteria with regard to a diagnosis of ARDS (Figure 1). Of these 192 patients, 164 did not meet any of the secondary exclusion criteria and were evaluated further (Figure 1). These patients were categorized into sivelestat (*n* = 87) and control (*n* = 77) groups, from which 31 propensity score-matched pairs and 329 IPTW patients (162 vs. 167) were generated. Number of patients (*n*) in the IPTW groups was an “estimated number” determined by weighting inversed probability (i.e., propensity score). Distributions of propensity scores in the unmatched, propensity score-matched, and IPTW groups are shown in Additional file 1: Figure S1.

Table 1 shows the baseline characteristics of the unmatched sivelestat and control groups (*n* = 164), IPTW group (*n* = 329), and propensity score-matched groups (*n* = 62). When the unmatched groups were compared, patients were more likely to receive sivelestat if they had a positive fluid balance and higher EVLWi on Day 0. After propensity score IPTW was performed, the baseline patient characteristics were well balanced between the groups. However, we found that the standardized difference of some variables in the propensity-matched groups exceeded 0.10, suggesting that these were not well balanced. Thus, we decided that it was not appropriate to perform further analysis using the matched pair approach. The median period of sivelestat use was 9 (quartile, 6) days.

**Table 1:** **Patient characteristics**

**Patient characteristic**	**Unadjusted groups**			**IPTW Groups**			**Matched groups**		
	**Sivelestat (** ***n*** **= 87)**	**Control (** ***n*** **= 77)**	**Standardized difference**	**Sivelestat (** ***n*** **= 162)**	**Control (** ***n*** **= 167)**	**Standardized difference**	**Sivelestat (** ***n*** **= 31)**	**Control (** ***n*** **= 31)**	**Standardized difference**
Age, year	66.1 (17.5)	65.9 (16.2)	0.01	67.7 (16.2)	69.0 (15.8)	−0.08	69.8 (15.6)	67.3 (15.3)	0.16
Male sex	57 (65.5)	50 (64.9)	0.01	104 (64.2)	114 (68.3)	−0.09	21 (67.7)	19 (61.3)	0.13
Academic hospital	49 (56.3)	48 (63.3)	−0.14	92 (56.8)	86 (51.8)	0.10	20 (64.5)	15 (48.4)	0.33
Hospital volume (cases)									
Low (1–10)	37 (42.5)	43 (55.8)	−0.27	73 (45.1)	70 (41.9)	0.06	15 (48.4)	14 (45.2)	0.06
Medium (11–20)	19 (21.8)	18 (23.4)	−0.04	32 (19.8)	34 (20.4)	−0.01	8 (25.8)	5 (16.1)	0.28
High (≥21)	31 (35.6)	16 (20.8)	0.33	57 (35.2)	63 (37.7)	−0.05	8 (25.8)	12 (38.7)	−0.28
Direct lung injury	54 (62.1)	44 (57.1)	0.10	94 (58.4)	104 (62.3)	−0.08	19 (61.3)	21 (67.7)	−0.13
APACHE II	22.2 (7.4)	23.5 (7.4)	−0.18	23.5 (7.3)	23.3 (7.5)	0.03	23.7 (5.5)	24.0 (7.6)	−0.05
SOFA score	10.1 (3.3)	11.2 (3.6)	−0.32	10.7 (3.3)	10.6 (3.7)	0.03	10.8 (3.3)	10.0 (3.2)	0.25
SIRS score	2.3 (1.0)	2.4 (1.2)	−0.09	2.4 (1.1)	2.4 (1.1)	0.00	2.4 (1.2)	2.5 (1.1)	−0.09
MAP, mmHg	75.5 (18.3)	77.2 (15.5)	−0.10	77.3 (19.1)	78.8 (17.2)	−0.08	75.8 (20.1)	73.9 (14.3)	0.11
CVP, mmHg	10.7 (5.4)	9.3 (5.2)	0.26	9.8 (5.2)	9.7 (5.1)	0.02	10.5 (4.7)	9.5 (5.8)	0.19
Fluid balance on Day 0, mL	2,239 (2,228)	1,498 (1,640)	0.38	1,915 (1,977)	1,861 (1,686)	0.03	1,495 (2,267)	1,673 (2,333)	−0.08
PaO_2_/FiO_2_	148.5 (73.1)	148.4 (65.6)	0	142.9 (70.2)	145.6 (63.9)	−0.04	141.7 (71.1)	128.5 (60.2)	0.20
PEEP, cm/H_2_O	8.4 (4.9)	8.3 (4.1)	0.02	8.2 (4.6)	8.1 (3.8)	0.02	8.1 (5.0)	8.8 (4.6)	−0.15
Cardiac index, L/m^2^	3.5 (1.4)	3.5 (1.2)	0	3.4 (1.4)	3.4 (1.1)	0.00	3.7 (1.5)	3.2 (1.0)	0.39
GEDI, mL/m^2^	836.9 (236.4)	784.2 (158.0)	0.26	809.9 (208.4)	805.2 (149.3)	0.03	805.9 (167.5)	761.9 (150.1)	0.28
EVLWi, mL/kg	20.0 (7.1)	17.2 (6.4)	0.41	19.1 (6.4)	19.4 (7.6)	−0.04	17.1 (6.2)	18.5 (7.1)	−0.21
PVPI	3.5 (1.5)	3.1 (1.2)	0.29	3.3 (1.3)	3.4 (1.3)	−0.08	3.0 (0.98)	3.5 (1.5)	−0.39
Corticosteroid use	38 (43.7)	26 (33.8)	0.20	62 (38.3)	69 (41.3)	−0.06	12 (41.9)	10 (32.3)	0.20
C*atecholamine* use	58 (66.7)	59 (76.6)	−0.22	117 (72.2)	113 (67.7)	0.10	23 (74.2)	23 (74.2)	0.00
Renal replacement therapy	25 (28.7)	18 (23.4)	0.12	45 (27.8)	50 (30.1)	−0.05	9 (29.0)	6 (19.4)	0.23
Diuretic use	43 (49.4)	38 (49.4)	0.00	75 (46.6)	84 (50.3)	−0.07	16 (51.6)	13 (41.9)	0.20
PMX use	12 (13.8)	6 (7.6)	0.20	18 (11.1)	14 (8.4)	0.09	4 (12.9)	1 (3.2)	0.36

Number of patients (*n*) in the IPTW groups was an estimated number determined by weighting inversed probability (propensity score).

*APACHE*, acute physiology and chronic health evaluation, *CVP*, central venous pressure, *EVLWi*, extravascular lung water index, *GEDI*, global end-diastolic volume index, *IPTW*, inverse probability of treatment weighting, *PEEP*, positive end-expiratory pressure, *PMX*, polymyxin B hemoperfusion, *PVPI*, pulmonary vascular permeability index, *SIRS*, systemic inflammatory response syndrome, *SOFA*, sequential organ failure assessment.

### Endpoints

The overall 28-day mortality was 31.1% (51/164). There was no significant difference in 28-day mortality between the study groups (sivelestat vs. control; unmatched: 29.9% vs. 32.5%; difference, −2.6%, 95% confidence interval (CI), −16.8 to 14.2; IPTW: 24.7% vs. 29.5%, difference, −4.8%, 95% CI, −14.4 to 9.6).

Although there was no difference in the number of VFDs in the sivelestat and control groups for unmatched patients (9.6 vs. 9.7 days; difference, 0.1, 95% CI, −3.0 to 3.1), there were significantly more VFDs in the sivelestat group than in the control group for the IPTW (10.7 vs. 8.4 days, difference, −2.3, 95% CI, −4.4 to −0.2).

## Discussion

This retrospective multi-institutional study did not identify any significant association between the 28-day mortality and sivelestat use. However, there may be a weak association between sivelestat use and an increased number of VFDs in ARDS patients with increased EVLW.

Although sivelestat has been approved and is currently used clinically in Japan and Korea, its effectiveness in ARDS patients remains controversial. Previous studies reported conflicting results [7-10,35,36,38,45,47,48,51]. While some reported that sivelestat administration was an independent predictor of survival and contributed to early improvements in oxygenation, early weaning from mechanical ventilation, and early discharge from the ICU [9,10,35,36,47,48], others reported little effect [51] or even negative effects [8].

The strength of the current study was that we took the EVLWi and PVPI into consideration during patient selection and the process used to estimate propensity scores. Previous studies suggested that diffuse alveolar damage resulted in significant accumulation of EVLW in patients with the early phase of ARDS [3,13,52], which led to severe respiratory failure and dependence on mechanical ventilation. Although it is difficult to evaluate the degree of lung injury quantitatively, introduction of the transpulmonary thermodilution technique has facilitated bedside evaluation of the EVLW with robust validation [14,18,25,27-29,53,54]. We recently validated the clinicopathological relationships between the EVLW and diffuse alveolar damage by conducting pathologic studies and a nationwide autopsy database study [18]. Recent studies showed that the EVLWi reflected the severity of lung injury and correlated with mortality in ARDS patients [15-17,55-57]. In the current study, only ARDS patients with an EVLWi of >10 mL/kg were included, consistent with previous related studies [19]. The normal EVLWi is approximately 7 mL/kg [27], and an EVLWi of >10 mL/kg represented the quantitative threshold for the diagnosis of ARDS [18]. Recent studies suggested that the EVLWi (and PVPI) may provide the most reliable characterization of ARDS, where the development of diffuse alveolar damage results in increased permeability and accumulation of water in the lungs [19,25]. Thus, EVLWi and PVPI provide key clinical insights into the underlying disease pathology.

The propensity score analysis approach is a powerful tool that attempts to construct a randomized experiment-like situation by comparing groups with similar characteristics without specifying the relationships between confounders and outcomes. In the current study, analysis of the baseline patient characteristics in the unmatched group (the overall study population) showed more sivelestat use in patients with increased EVLWi and positive fluid balance, which are both known to affect outcome in ARDS patients [2,15]. We therefore believe that these variables need to be balanced when evaluating the effect of sivelestat on ARDS. In our study, factors that had the potential to affect mortality, or were known to affect mortality in patients with ARDS, were successfully balanced in the IPTW analysis. Our results suggested that administration of sivelestat does not influence 28-day mortality. On the other hand, the IPTW analysis suggested that ARDS patients who were prescribed sivelestat had more VFDs than similar patients who were not. Although we could not draw any robust conclusions regarding the effect of sivelestat in the current retrospective analysis, these results can be considered as hypothesis-generating. Thus, further large prospective trials, considering EVLWi in the entry criteria, are required to confirm the current results.

This study had several limitations. First, the study was conducted retrospectively; it was not randomized or blinded, and the decision regarding administration of sivelestat was made by the doctors in charge of each of the 23 participating institutions. Although propensity score methods were used to adjust for differences in baseline characteristics and disease severity, bias could still be present in the form of confounders that were not measured. Second, even though 164 patients were evaluated in the current study, the nearest-neighbor matching method only identified 31 pairs for one-to-one matched analysis. As a result, the standardized difference of several variables in the propensity-matched groups exceeded 0.10, suggesting that these were not well balanced. This may be due to the small sample size (31 pairs). In general, pair matching on the propensity score requires that the number of untreated subjects be larger (and preferably substantially larger) than the number of treated subjects [31]. Thus, matching will not perform well when the two samples are of approximately equal size or when the number of treated subjects is larger than the number of untreated subjects [31]. On the other hand, IPTW methods do not suffer from this limitation [31]. One of the strengths of the IPTW method is that data from all of the patients were used. On the other hand, treated subjects with a very low propensity score or untreated subjects with a high propensity score will have large weights, as shown in Additional file 1: Figure S1. This might be a significant concern if the study group was heterogeneous. However, in the current study, we tried our best to select a homogeneous patient group by utilizing strict inclusion and exclusion criteria (i.e., even the control group had the potential to receive sivelestat): all patients were mechanically ventilated in the tertiary care hospital ICU for >48 h due to acute respiratory failure, PaO_2_/FiO_2_ ratio of ≤300 mmHg, EVLWi of >10 mL/kg, and diagnosed as permeability pulmonary edema. Third, patients who were not administered sivelestat on Day 0 were excluded from the current analysis to exclude the possibility of immortal-time bias [58]. Thus, we could not investigate the effect of sivelestat on the late phase of ARDS.

## Conclusions

Although there was no significant association between 28-day mortality and administration of sivelestat, the treatment may have the potential to increase the number of VFDs in ARDS patients with increased EVLW. Prospective randomized controlled studies are required to confirm the results of the current study.

## Additional file

Additional file 1: Figure S1. Distribution of propensity scores in the unmatched, propensity score-matched, and propensity score inverse probability of treatment weighting (IPTW) groups.

## Abbreviations

APACHE: Acute physiology and chronic health evaluation
ARDS: Acute respiratory distress syndrome
CVP: Central venous pressure
EVLW: Extravascular lung water
EVLWi: Extravascular lung water index
NE: Neutrophil elastase
PEEP: Positive end-expiratory pressure
PVPI: Pulmonary vascular permeability index
SOFA: Sequential organ failure assessment
CI: Confidence interval
