# Supplementary material for: Effect of a selective neutrophil elastase inhibitor on mortality and ventilator-free days in patients with increased extravascular lung water: a post hoc analysis of the PiCCO Pulmonary Edema Study
Source: J Intensive Care. 2014 Dec 31;2(1):67. doi: 10.1186/s40560-014-0067-y (PMC4336272; doi:10.1186/s40560-014-0067-y)

**Additional File 1**

**Figure S1.**

**Distribution of propensity scores in the unmatched, propensity score matched, and propensity score inverse probability of treatment weighting (IPTW) groups**

**Fig. S1**


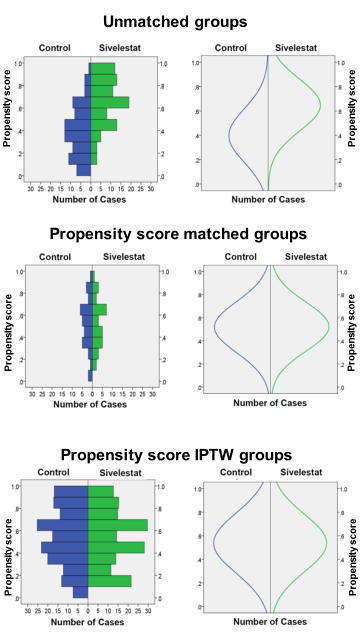

Supplement: Additional file 1: Figure S1. — Distribution of propensity scores in the unmatched, propensity score-matched, and propensity score inverse probability of treatment weighting (IPTW) groups. [file 40560_2014_67_MOESM1_ESM.docx]
